# Using Social Media to Perform Local Influenza Surveillance in an Inner-City Hospital: A Retrospective Observational Study

**DOI:** 10.2196/publichealth.4472

**Published:** 2015-05-29

**Authors:** David Andre Broniatowski, Mark Dredze, Michael J Paul, Andrea Dugas

**Affiliations:** ^1^ Department of Engineering Management and Systems Engineering The George Washington University Washington, DC United States; ^2^ Human Language Technology Center of Excellence Johns Hopkins University Baltimore, MD United States; ^3^ Department of Computer Science Johns Hopkins University Baltimore, MD United States; ^4^ Department of Emergency Medicine Johns Hopkins University Baltimore, MD United States

**Keywords:** Web mining, social computing, time series analysis

## Abstract

**Background:**

Public health officials and policy makers in the United States expend significant resources at the national, state, county, and city levels to measure the rate of influenza infection. These individuals rely on influenza infection rate information to make important decisions during the course of an influenza season driving vaccination campaigns, clinical guidelines, and medical staffing. Web and social media data sources have emerged as attractive alternatives to supplement existing practices. While traditional surveillance methods take 1-2 weeks, and significant labor, to produce an infection estimate in each locale, web and social media data are available in near real-time for a broad range of locations.

**Objective:**

The objective of this study was to analyze the efficacy of flu surveillance from combining data from the websites Google Flu Trends and HealthTweets at the local level. We considered both emergency department influenza-like illness cases and laboratory-confirmed influenza cases for a single hospital in the City of Baltimore.

**Methods:**

This was a retrospective observational study comparing estimates of influenza activity of Google Flu Trends and Twitter to actual counts of individuals with laboratory-confirmed influenza, and counts of individuals presenting to the emergency department with influenza-like illness cases. Data were collected from November 20, 2011 through March 16, 2014. Each parameter was evaluated on the municipal, regional, and national scale. We examined the utility of social media data for tracking actual influenza infection at the municipal, state, and national levels. Specifically, we compared the efficacy of Twitter and Google Flu Trends data.

**Results:**

We found that municipal-level Twitter data was more effective than regional and national data when tracking actual influenza infection rates in a Baltimore inner-city hospital. When combined, national-level Twitter and Google Flu Trends data outperformed each data source individually. In addition, influenza-like illness data at all levels of geographic granularity were best predicted by national Google Flu Trends data.

**Conclusions:**

In order to overcome sensitivity to transient events, such as the news cycle, the best-fitting Google Flu Trends model relies on a 4-week moving average, suggesting that it may also be sacrificing sensitivity to transient fluctuations in influenza infection to achieve predictive power. Implications for influenza forecasting are discussed in this report.

## Introduction

**Figure 1:**
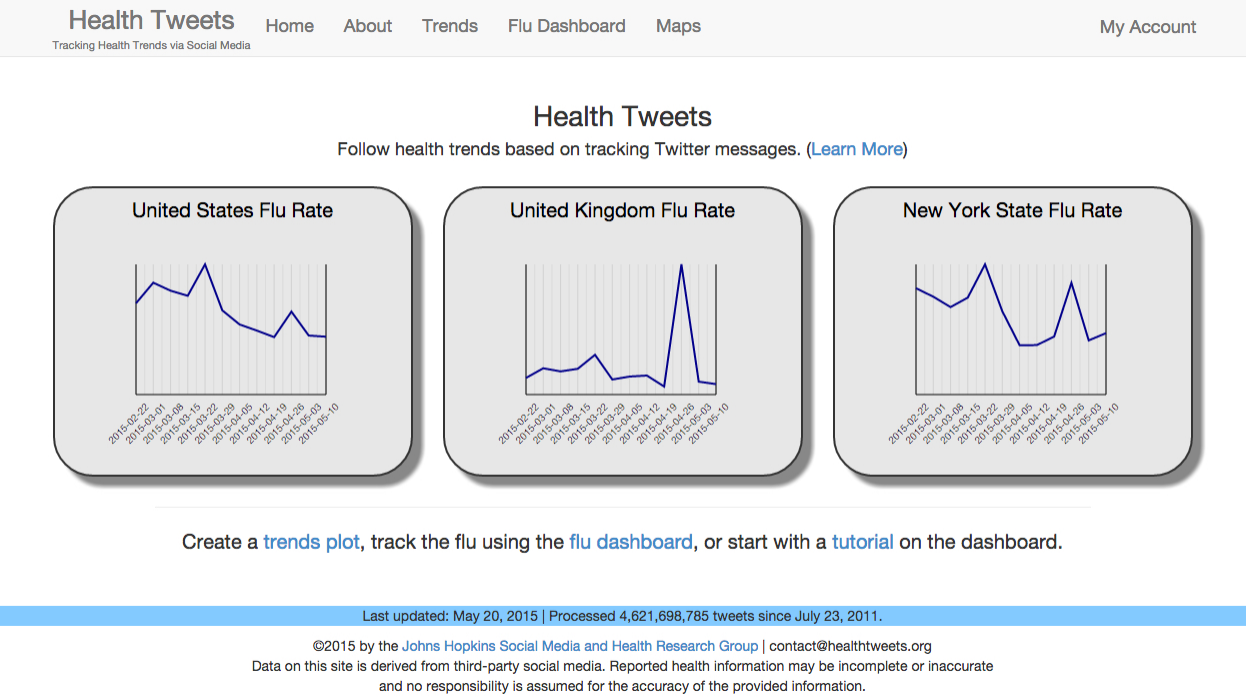
Screenshot of HealthTweets.

Public health officials and policy makers rely on influenza infection rate information to make important decisions during the course of an influenza season. Whereas influenza surveillance has traditionally been conducted using laboratory data, hospitalizations, and physician visits for influenza-like illness (ILI), web and social media data sources have emerged as attractive alternatives to supplement existing practices. While traditional surveillance methods take 1-2 weeks, and significant labor, to produce an infection estimate in each locale, web and social media data are available in near real-time for a broad range of locations. Studies have demonstrated that web queries [1-3], Twitter messages [4-12], and other sources (eg, Wikipedia [13], mobile app reporting [14]) may be productively mined for influenza surveillance data. New resources like Google Flu Trends [1], HealthTweets [15,16](Figure 1), and Flu Near You [14] deliver near-real time estimates of infection rates.

However, few have examined the efficacy of local surveillance [12,17,18]. In this study, we analyzed the efficacy of local flu surveillance from Google Flu Trends and HealthTweets. Whereas previous studies that considered either Google or Twitter in isolation, we evaluated multiple trends available from both. Furthermore, instead of restricting our study to hospitals designated as ILI sentinels, or emergency department ILI rates, we considered both emergency department ILI and laboratory-confirmed influenza cases for a single hospital in the city of Baltimore. This enabled us to evaluate the impact on specific care centers when making influenza response decisions, such as staffing and resource allocation.

## Methods

### Study Population and Setting

This was a retrospective observational study comparing estimates of influenza activity from Google flu trends and Twitter to actual counts of individuals with laboratory-confirmed influenza, and counts of individuals presenting to the emergency department with ILI. Each parameter was evaluated on the municipal, regional, and national scale.

### Data Collection and Methods of Measurement

Data were collected from November 20, 2011 through March 16, 2014. All measurements were recorded weekly to allow for direct comparison between data sources. Following the Centers for Disease Control (CDC) Convention, each week summed the data points from Sunday through the following Saturday. The number of municipal- (city) level subjects was estimated by evaluating the number of patients presenting to an urban academic emergency department in Baltimore, Maryland with an annual volume of over 60,000 adult and 24,000 pediatric visits. The number of confirmed influenza cases was determined by summing the number of emergency department visits with laboratory-confirmed influenza that occurred during each week. Similarly, the number of patients with ILI was determined by summing the number of emergency department patients who reported fever with cough or sore throat each week. Regional data were collected via the CDC surveillance reports for health and Human Services (HHS) Region 3, including both the percentage of patients reporting ILI and the percentage of tests positive for influenza. National data were collected from the CDC surveillance report of the nationwide percentage of patients reporting ILI and the total percentage of patients testing positive for influenza.

Google Flu Trends data for the United States, the state of Maryland, and the city of Baltimore were downloaded directly from the Google Flu Trends website [19]. Twitter data for the same three locations was obtained from the HealthTweets website [15], an online platform for public health surveillance aimed at sharing the latest research results on Twitter data with the scientific community and public officials. The underlying data were generated using a sequence of supervised machine-learning algorithms [10,12], namely logistic regression classifiers, the first of which identified tweets that were relevant to health. Next, tweets that were about influenza were isolated. The final classifier separated tweets that were about reported influenza infection from those that only reported awareness of the flu. The tweets indicating influenza infection constituted our dataset. Message locations were identified using Carmen [20], a software package that infers tweet locations using Global Positioning System (GPS) coordinates and self-reported locations from the free text of the user biographic profiles.

### Statistical Analysis

**Figure 2:**
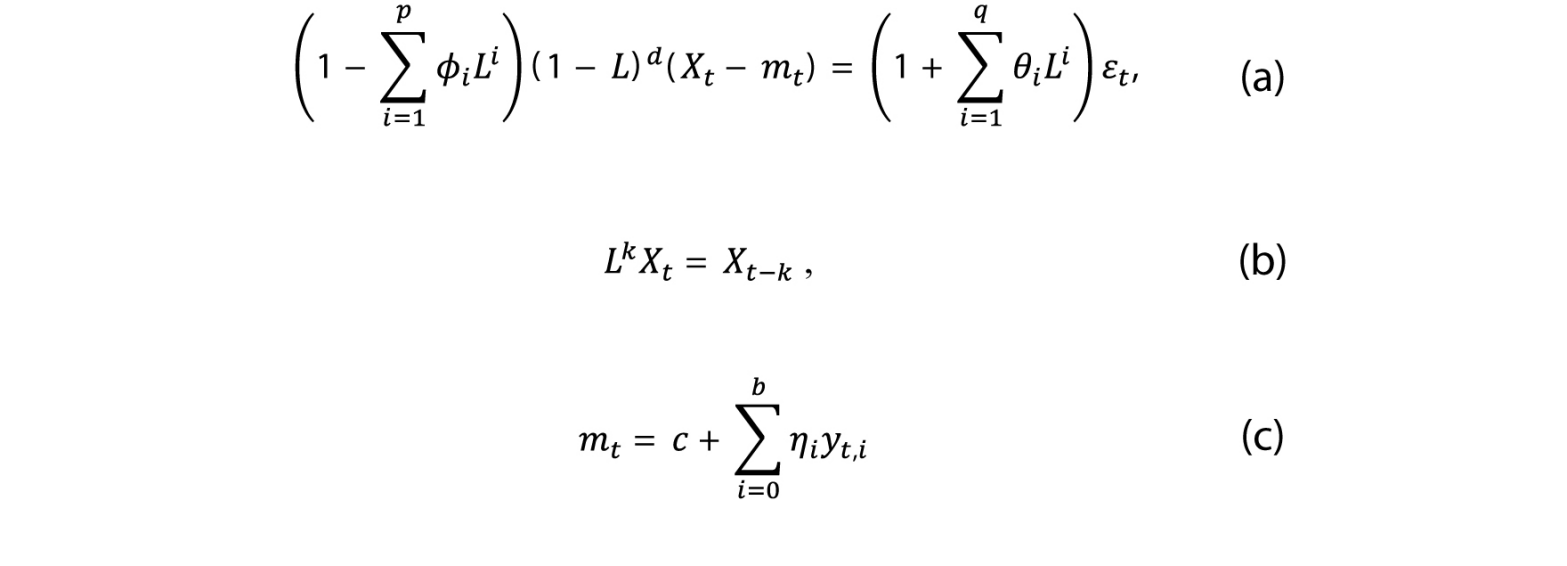
Equations defining the ARIMAX model.

Data were analyzed by evaluating weekly trends over time using the Box-Jenkins procedure [21] applied to each data source (influenza tests at our medical center, ILI at our medical center, % reported flu cases in HHS region 3 and the USA, and % reported ILI in HHS region 3 and the USA) in order to control for autocorrelation in the corresponding time series. We next fit an autoregressive integrated moving average model with exogenous covariates (ARIMAX) to each data time series, X_t_, where p, d, and q, are the respective autoregressive, differencing, and moving average orders of the model (Figure 2 , part a). The φ_i_and θ_i_are the autoregressive and moving average parameters, respectively, ε_t_is a normally distributed error term with a mean of 0, L is a lag operator defined as in Figure 2 , part b, and m_t_is defined as in Figure 2 , part c, where y_t_is a series of predictors (eg, Twitter and/or Google Flu Trends data), the η_i_are a series of predictor weights, and b is the total number of predictor time series.

We chose the autoregressive, differencing, and moving average terms of each model that minimized each its Aikake Information Criterion (AIC) subject to the constraint that each model used the same degree of differencing for each data source. This constraint was imposed to enable comparison across social media predictors (ie, Twitter, Google Flu Trends, or both). All statistics were conducted using the R Project for Statistical Computing, version 3.0.2 (The R Foundation for Statistical Computing). Specifically, we used the "arima()” function in the forecast package [22]. Parameter selection was informed by the “auto.arima()” function, using the Hyndman and Khandakar algorithm [23]. Deviations from the algorithm’s output were then examined by hand and parameters that deviated from algorithm output were chosen if they minimized AIC.

## Results

**Figure 3:**
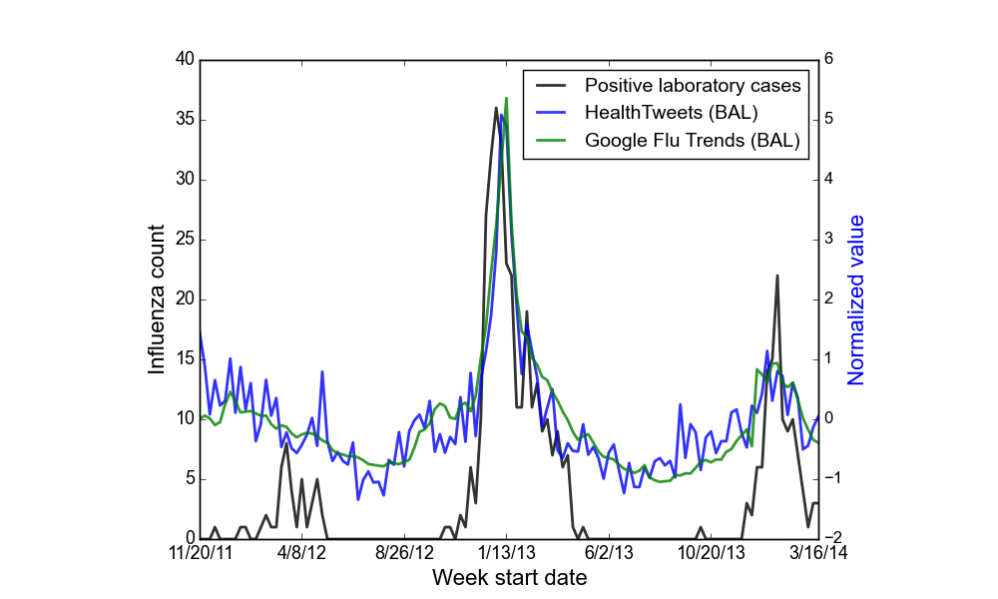
Plot of weekly confirmed influenza cases (right axis) as compared to standardized Baltimore social media data (left axis).

Table 1 summarizes the results of each ARIMA model incorporating Twitter and Google Flu Trends data. Our results show that Baltimore-area Twitter data provided a better estimate of actual influenza cases reported in the Baltimore metropolitan area when compared to state- and national-level Twitter data (see Figure 3). Furthermore, a combination of Twitter and Google Flu Trends data sources outperformed either Twitter or Google Flu Trends individually when predicting actual influenza outbreaks at municipal and regional levels.

**Table 1:** Log-likelihood (AIC^a^) for each surveillance method.

		Laboratory-confirmed influenza			Influenza like illness (ILI)		
		City	Region	US	City	Region	US
**Twitter** ^b^							
	US^c^	-311 (627)^0,1,0e^	-317^g^(653)^5,1,3^	-235^g^(484)^0,1,5^	-502^g^(1009)^0,2,1^	-66^g^(143)^0,1,0^	-27^g^(61)^1,1,1^
	MD^d^	-310 (624)^0,1,0^	-321 (661)^5,1,3^	-236 (486)^0,1,5^	-503 (1012)^0,1,0^	-70 (144)^0,1,0^	-30 (68)^1,1,1^
	Baltimore	-308^g^(620)^0,1,0^	-323 (666)^5,1,3^	-235 (484)^0,1,5^	-504 (1013)^0,2,1^	-74 (158)^0,1,3^	-32 (74)^1,1,1^
**Google Flu Trends**							
	US	-291^g^(596)^1,1,4^	-313^g^(648)^5,1,4^	-230^f,g^(475)^0,1,5^	-494^f,g^(1002)^1,2,4^	-49^f,g^(110)^0,1,4^	-1^f,g^(15)^1,1,4^
	MD	-299 (612)^1,1,4^	-318 (656)^5,1,3^	-236 (486)^0,1,5^	-498 (1010)^1,2,4^	-58 (129)^0,1,4^	-27 (61)^1,1,1^
	Baltimore	-295 (604)^1,1,4^	-320 (660)^5,1,3^	-236 (486)^0,1,5^	-495 (1005)^1,2,4^	-60 (132)^0,1,4^	-23 (56)^1,1,2^
**Both**							
	US	-289^f,g^(594)^1,1,4^	-312^f,g^(646)^5,1,3^	-230^g^(477)^0,1,5^	-495^g^(1003)^0,1,4^	-49^g^(112)^0,1,4^	-0^g^(17)^1,1,4^
	MD	-299 (613)^1,14^	-318 (657)^5,1,3^	-235 (485)^0,1,5^	-498 (1011)^1,2,4^	-58 (130)^0,1,4^	-27 (68)^1,1,1^
	Baltimore	-294 (604)^1,1,4^	-319 (659)^5,1,3^	-235 (486)^0,1,5^	-500 (1007)^0,2,1^	-60 (134)^0,1,4^	-22 (55)^1,1,2^

^*a*^AIC=Aikake Information Criterion

^b^Twitter data from the HealthTweets website.

^c^US=United States

^d^MD=Maryland

^e^Superscript numerals indicate the autoregressive order, the order of differencing, and the moving average order, respectively. Models were chosen to minimize AIC, guided by examinations of autocorrelation and partial autocorrelation values.

^f^The best predictor across all data sources.

^g^The best predictor within each data source (HealthTweets website, Google, or a linear combination of both).

When directly comparing models that rely only on one data source (ie, Twitter or Google Flu Trends but not both), we found that the best-fitting Twitter models were simple whereas the best-fitting Google Flu Trends models generally required more parameters. For example, at the municipal level, the best-fitting Twitter model did not require any autoregressive or moving average terms, whereas the best-fitting Google Flu Trends model required a 4-week moving average of Google Flu Trends data and an autoregressive term. In general, these more complex Google Flu Trends models outperformed the best-fitting Twitter models. Although these Google Flu Trends models were significantly more complex (ie, one must fit more parameters), they had a lower AIC, indicating that they were also more informative.

## Discussion

### Principal Findings

Consistent with prior work [18], we found that national-level Google Flu Trends data may be used to track actual influenza cases in the Baltimore area. The fact that a combination of Twitter and Google Flu Trends data at the national (US) level outperformed all other data sources for local and regional confirmed influenza cases indicates that these data sources are not redundant and that Twitter data are contributing information useful to influenza surveillance that are not captured by the corresponding Google Flu Trends data.

### Comparison With Prior Work

**Figure 4:**
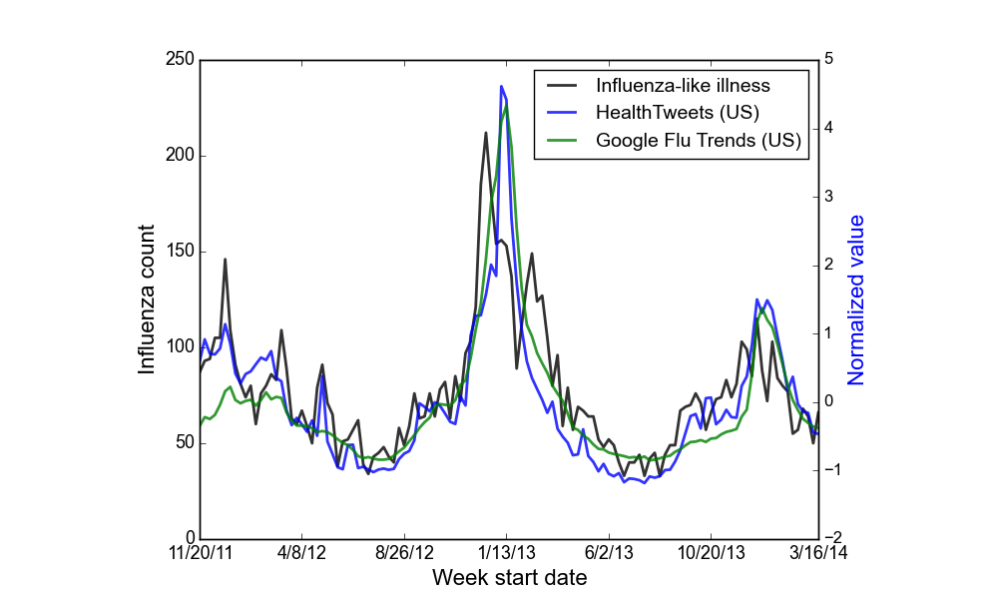
Plot of weekly influenza-like illness cases (right axis) as compared to standardized US social media data (left axis).

Whereas prior work using Google Flu Trends data has largely focused on US ILI data, we extended this finding to multiple levels of geographic granularity by examining social media surveillance at the regional and city levels as well. We found that US Google Flu Trends data best explained ILI rates at all levels (including the municipal level, see Figure 4). This contrasts with prior research, which found that Google Flu Trends data conflated signals of influenza awareness (eg, media attention) with signals of actual infection - overestimating the flu season’s peak prevalence. In addition, this prior work found that there was insufficient control for temporal autocorrelation and a lack of analysis of Google Flu Trends data at local, rather than national, levels [24].

In this study, we controlled for autocorrelation and exogenous temporal factors using an ARIMAX model. The improved performance of this model might be an indication that the 4-week moving average terms are smoothing out fluctuations due to the news cycle. Nevertheless, because Google Flu Trends data do not explicitly differentiate between signals of influenza awareness and actual infection, this relatively complicated model may buy accuracy at the cost of sensitivity to transient phenomena. Thus, temporary spikes in media coverage are smoothed out, but so would temporary spikes in influenza infection.

Elsewhere, we have shown that our Twitter data overcome the limitations identified in prior Google Flu Trends studies by filtering out signals of influenza awareness from signals of actual infection and enabling analysis at multiple levels of geographic granularity [12,25]. Furthermore, the fact that the Twitter model is more lightweight means that it is more able to correctly track transient increases in infection when they occur [12]. Finally, municipal-level Twitter data provided a better account of actual influenza cases in Baltimore than did state- or national- level data. This finding is consistent with prior work [12] showing that local Twitter data does contribute information that is useful for municipal surveillance. In contrast, state- and local-level Google Flu Trends data did not improve surveillance when compared to national GFT data.

### Limitations

One limitation of our approach is that it only relies upon one municipality. Furthermore, our analysis only examined three seasons of influenza data, one of which (the 2012-2013 season) is known to have been anomalous. Future work should therefore focus on incorporating data from multiple influenza seasons.

### Conclusions

Overall, our results motivate the need for future work examining how social media may be used to track measures relevant to influenza surveillance in multiple different locations and seasons.

## Abbreviations

AIC: Aikake information criterion
ARIMA: Autoregressive integrated moving average
CDC: Centers for Disease Control
HHS: Health and Human Systems
ILI: Influenza-like illness
